# Cost Analysis of an Intervention to Prevent Methicillin-Resistant *Staphylococcus Aureus* (MRSA) Transmission

**DOI:** 10.1371/journal.pone.0138999

**Published:** 2015-09-25

**Authors:** Michal Chowers, Yehuda Carmeli, Pnina Shitrit, Asher Elhayany, Keren Geffen

**Affiliations:** 1 Infectious Diseases Unit, Meir Medical Center, Kfar Saba, Israel; 2 Sackler Faculty of Medicine, Tel Aviv University, Ramat Aviv, Israel; 3 National Center for Infection and Antibiotic Resistance Control, Tel Aviv Medical Center, Tel Aviv, Israel; 4 Department of Management, Bar Ilan University, Ramat Gan, Israel; 5 Department of economics, Meir Medical Center, Kfar Saba, Israel; Columbia University, UNITED STATES

## Abstract

**Introduction:**

Our objective was to assess the cost implications of a vertical MRSA prevention program that led to a reduction in MRSA bacteremia.

**Methods:**

We performed a matched historical cohort study and cost analysis in a single hospital in Israel for the years 2005-2011. The cost of MRSA bacteremia was calculated as total hospital cost for patients admitted with bacteremia and for patients with hospital-acquired bacteremia, the difference in cost compared to matched controls. The cost of prevention was calculated as the sum of the cost of microbiology tests, single-use equipment used for patients in isolation, and infection control personnel.

**Results:**

An average of 20,000 patients were screened yearly. The cost of prevention was $208,100 per year, with the major contributor being laboratory cost. We calculated that our intervention averted 34 cases of bacteremia yearly: 17 presenting on admission and 17 acquired in the hospital. The average cost of a case admitted with bacteremia was $14,500, and the net cost attributable to nosocomial bacteremia was $9,400. Antibiotics contributed only 0.4% of the total disease management cost. When the annual cost of averted cases of bacteremia and that of prevention were compared, the intervention resulted in annual cost savings of $199,600.

**Conclusions:**

A vertical MRSA prevention program targeted at high-risk patients, which was highly effective in preventing bacteremia, is cost saving. These results suggest that allocating resources to targeted prevention efforts might be beneficial even in a single institution in a high incidence country.

## Introduction

Bacteremia caused by methicillin-resistant *S*. *aureus* (MRSA) is associated with increased mortality and morbidity, leading to high healthcare expenditures. Colonized patients are 22 times more likely to develop an infection by MRSA than those who are not identified as carriers.[1] Colonization pressure, i.e., the proportion of patients colonized with MRSA in a health care facility, is one of the most important factors predicting whether a patient will acquire MRSA.[2] Without active screening, the majority of MRSA carriers remain unknown. Thus, screening for MRSA carriage is a pillar of targeted MRSA prevention programs. Although still controversial due to the lack of high quality data and contradictory findings from different studies,[3,4] screening for MRSA is recommended by the Society for Healthcare Epidemiology of America.[5] The debate over the relative effectiveness and cost effectiveness of vertical versus horizontal preventive measures is ongoing. [6,7]

In Israel, 35% of invasive *S*. *aureus* isolates are resistant to Methicillin.[8] MRSA screening is not widely performed. This partly reflects Israel's limited healthcare resources, the paucity of single patient rooms for isolation, the shortage of infection control personnel, and the lack of local data on the cost of MRSA infections and targeted MRSA prevention interventions.

In the mid-2000s, a program of targeted active surveillance for MRSA was instituted in our hospital.[9] The successful implementation of this program led to a sustained 70% reduction in hospital-acquired and hospital related MRSA bacteremia.[10]

The objective of this study was to compare the cost of the targeted, MRSA screening and prevention activities to the cost of averted MRSA bloodstream infections (BSI). The cost analysis was done from a hospital perspective.

The hypothesis was that the intervention to decrease MRSA bacteremia, would be cost saving.

## Methods

### Setting and Patient Population

Meir Medical Center is an academic hospital with 742 beds and approximately 60,000 admissions per year. It is the only hospital serving the south of the Sharon area in the center of Israel; thus, most readmissions are to the same hospital. The study population included adults (>18) hospitalized during 2005 through 2011. Of note, a prevention intervention started in July 2003 and was sustained through 2011.

Study design: A single center, matched, historical cohort study and cost analysis.

### Intervention

The details of the prevention program have been published elsewhere.[9,10] Briefly, the main activities were as follows: Patients at high risk for MRSA carriage (i.e. hospitalized in the previous month or transferred from another hospital or long-term care facility, on long-term hemodialysis, or with a history of MRSA carriage) were screened by nasal swab upon admission. Patients found to be MRSA carriers were placed in contact isolation and gloves and gowns were used for all patient care. Eradication treatment with nasal mupirocin and chlorhexidine body wash was used during the first year of the intervention, but was later abandoned when we observed increasing resistance to mupirocin. An infection control nurse monitored compliance with screening guidelines and isolation measures.

### Calculation of the Cost of Prevention

The cost of prevention was calculated as the sum of the cost of the swabs and microbiology tests, the salary of the infection control team (physician and nurse), the salary for nursing time required to take the swabs, and the cost of gloves and gowns used for contact isolation. The cost of the microbiology tests included cost of technician and perishables. We did not include the cost of a private room: because of the paucity of single-bed rooms, the majority of patients were isolated in multi-bed rooms.

### Calculation of the Cost of Infection

For patients admitted with MRSA bacteremia, we considered the entire hospital cost to be related to the bacteremia. To calculate the bacteremia-associated cost for patients who acquired the infection during hospitalization, we matched each of these patients with two control patients who were hospitalized for at least the same duration as cases (until the bacteremia developed) in the same division (medicine, surgery or intensive care). The control patients were chosen randomly from all eligible patients using a random number assignment. We elected the matched design (to control for confounding by design) rather than the propensity score methods due to ease of understanding and because the two major determinants of cost are well known: length of stay before the event and the type of unit where the patient is hospitalized. The two methods have been shown to yield comparable results in these settings. [11] The excess cost of the cases compared with the matched controls was calculated after controlling for confounders. The following data were collected: age, sex, Charlson Comorbidity Index score, length of stay (LOS) by department (medical vs. surgical), laboratory work, imaging, antibiotic therapy, treatments such as dialysis, and procedures. To account for readmissions, data were collected for the index hospitalization as well as for repeat hospitalization related to the bacteremia within six months.

Hospital costs consist of fixed costs (70% of the average daily hospital cost) and variable costs. The per-patient variable cost was calculated by including the cost of drugs, procedures, imaging, and laboratory tests. Costs were those paid by the hospital and not charges billed by the hospital.

Surgical procedures may dramatically affect the variable costs and often were not related to the infection. Therefore, in order to eliminate this bias, only surgical procedures that were directly related to the infection were included in the variable cost, e.g., valve replacement for endocarditis or drainage of an epidural abscess.

In a previous study, [10] we determined that our intervention reduced the incidence of all hospital related MRSA bacteremia by 70%. Hospital related bacteremia was either bacteremia developing >48 hours after hospitalization or patients admitted with bacteremia, but who were hospitalized in our hospital during the previous year (community-onset, hospital-related MRSA bacteremia (CO-HR)). [10] Of note, among those with community-onset MRSA bacteremia, 68% were hospitalized in our hospital in the previous year. The other 32% of patients admitted with bacteremia were not hospitalized in the year preceding the bacteremia or were hospitalized in another hospital, and thus were not subjected to our prevention activities.

The total cost averted by the intervention was calculated as follows:

$$FC=\frac{\left(A+(AvgB-AvgC)Ns\right)}{0.3}0.7$$

FC- Total cost averted in 2005–2011

A-Cost of all patients admitted with MRSA BSI*68%

avgB- average cost of a patient who acquired MRSA BSI during hospitalization

avgC- average cost of controls

Ns- number of patients who acquired infection MRSA BSI during hospitalization

## Statistics

All analyses were performed using Stata V. 12 software (StataCorp, College Station, Texas, USA). For cases of hospital-acquired MRSA bacteremia and their matched controls, three matched analyses were performed: linear regressions with an absorbed variable to examine the outcomes of log-transformed cost, length of hospital stay and fixed-effect (conditional) logistic regression to examine the outcome of mortality. To control for confounders, demographics, exact duration of hospital stay, and comorbid conditions expressed by the Charlson score were examined. All variables with a P-value <0.05 in univariate analysis were eligible for inclusion in the multivariate model. All variables were also examined for effect modification (>20% change in the beta coefficient).

### Ethics

The study was approved by the Meir Medical Center Ethics Committee. Written informed consent was not required. Patient records were de-identified prior to analysis.

## Results

### MRSA-Directed Prevention Program and Its Annual Cost

**Fig 1:**
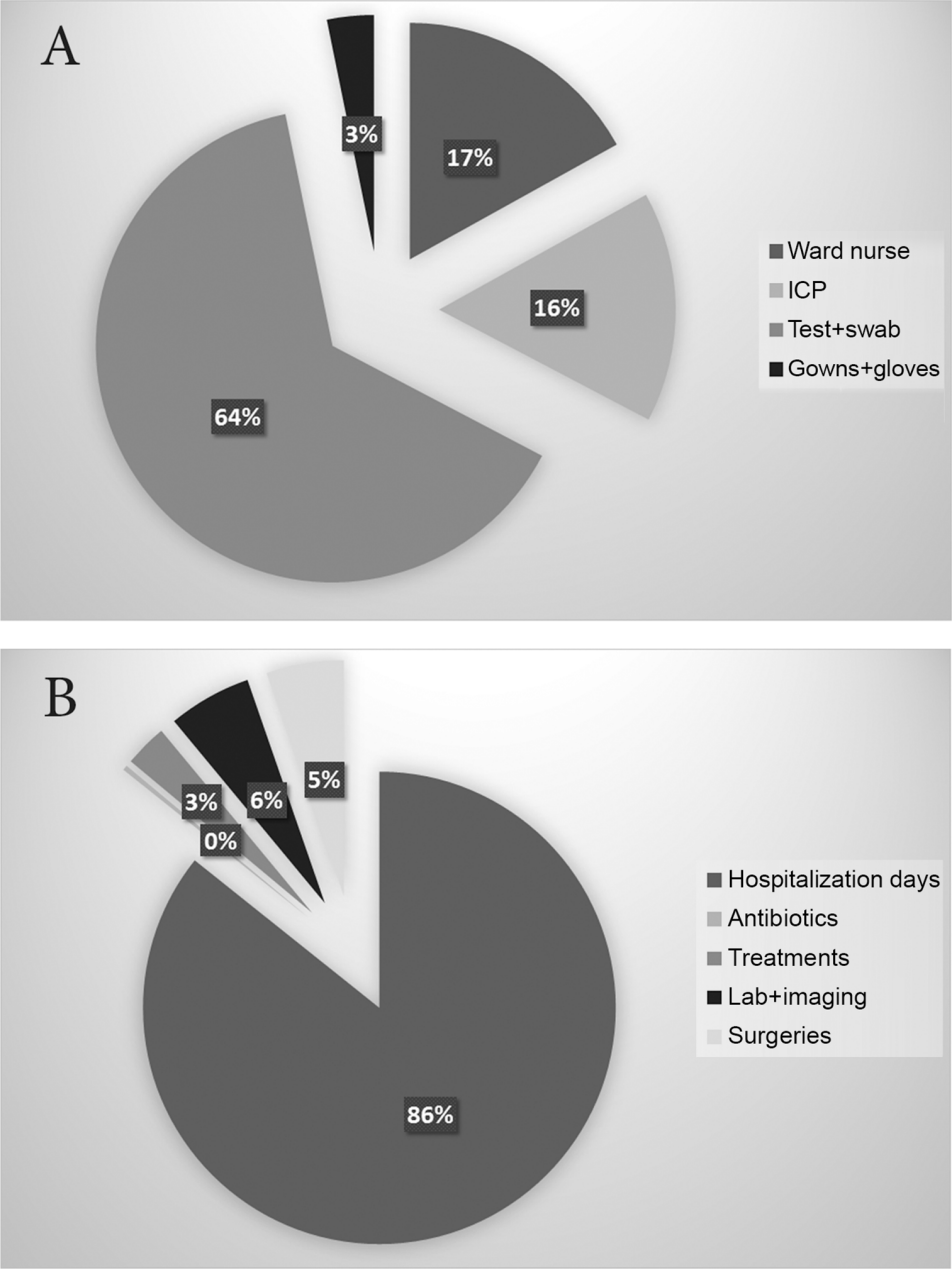
A: Components of the cost of MRSA prevention (ICP- infection control practitioner). B: Components of the cost of hospitalization of patients with MRSA bacteremia.

During the seven-year study, a total of 143,446 MRSA screening tests were performed. [10] The cost of prevention included the salary of a 0.4 full-time equivalent infection control nurse, the salary of a 0.1 full time equivalent infection control physician, the cost of 20,450 swabs and MRSA tests performed yearly, the cost of salary of five minutes nurse time for each swab taken and gloves and gowns used for contact isolation of an average of eight patients each day. These costs total $208,100 per year (Fig 1A); laboratory tests were the major component of the cost (64%).

### The Clinical Outcomes and Cost Associated with MRSA Bacteremia

From 2005 through 2011, 126 MRSA bacteremia cases were identified. Seventy-three patients were admitted with the infection and 53 developed bacteremia during hospitalization. To the latter group, i.e., cases with hospital-acquired MRSA bacteremia, 101 control patients were matched. Characteristics of the three groups are displayed in Table 1.

**Table 1:** Patient characteristics and outcomes.

	BSI on admission n = 73	Hospital-acquired BSI n = 53	Control n = 101	P value (hospital acquired vs control)
Age mean (SD)	72.5 (14.8)	72.8 (15.5)	71.1 (17.5)	0.54
Sex (female)	34 (46.6%)	26 (49%)	58 (58%)	0.32
Charlson score median (range)	4.0 (0–21)	2.0 (0.17)	4.0 (0–13)	0.58
LOS median (range)	20.0 (0–113)	21 (1–107)	6 (1–143)	<0.001
Mortality in hospital	26 (35.6%)	27 (51%)	16 (16%)	<0.001
Mortality within 72 hours of BSI	13 (17.8%)	10 (18.9%)		
Cost/patient ($) mean (SD)	14,300 (12,488)	14,900 (14,137)	5,600 (10,476)	<0.001
Cost/patient surviving> 72h after BSI ($) mean (SD)	16,600 (12,136)	18,500 (13,615)	5600 (10476)	<0.001

LOS-length of stay, BSI- Blood stream infection

In-hospital mortality among patients with MRSA bacteremia was high; 36% of those admitted with MRSA BSI and 51% with hospital-acquired BSI died. Among controls, 16% died during hospitalization (p<0.001).

Among patients with community-acquired or hospital-acquired BSI, a large variability in LOS was observed. Patients admitted with bacteremia were hospitalized for a mean of 19 days. Hospital-acquired bacteremia appeared at a mean of 17 days after admission. Despite the fact that 20% of cases with hospital- acquired BSI died within 72 hours of infection, and despite matching by length of stay up until BSI onset (in cases), their average length of stay was considerably longer than that of their matched controls (38.8 vs. 28 days, p = 0.05) (Table 1).

Fig 1B illustrates the distribution of the total cost of MRSA BSI. The main contributors were fixed costs (86%), while antibiotics constituted only 0.4% of the cost. Laboratory tests, surgeries and treatments such as dialysis each comprised only 3–5% of hospitalization costs.

The average cost of a case admitted with bacteremia was $14,500 and the average cost of a case that developed BSI during hospitalization was $14,900. We found a significant gender effect on the cost which we corrected for by analysis in the final cost. The average cost for a male who developed BSI during hospitalization was $11,700 vs. $18,100 for a female (p = 0.54), while the average cost per male control was $7,000 vs. $4,400 for a female control (p = 0.02). The unadjusted difference in cost between cases with hospital-acquired MRSA bacteremia and matched controls was $9,300 (Table 1). After adjusting for gender, the net cost attributable to hospital-acquired MRSA bacteremia was $9,400.

As noted before, a considerable percentage of cases died during hospitalization, some within 72 hours of bacteremia onset. The increased mortality led to a reduced length of stay and thus a decrease in average cost. A cost calculation that included only patients who survived at least 72 hours after bacteremia onset resulted in higher costs; $16,600 for patients admitted with bacteremia and $18,500 for patients with hospital-acquired bacteremia. The difference in gender unadjusted cost between cases with hospital-acquired MRSA bacteremia and matched controls (i.e., the added cost attributable to the bacteremia) was $13,000.

We calculated the cost averted by the prevention of MRSA bacteremia as follows: During the seven years studied, 126 patients were treated for MRSA bacteremia. Of the 73 patients admitted with bacteremia, 68% were related to our hospital and thus were subjected to our prevention program; i.e., over a seven year period 53 nosocomial cases and 50 CO-HR, with a calculated per-year cost of $174,700.

Assuming a sustained 70% decrease in the number of MRSA bacteremia cases because of the intervention, we estimated that 240 MRSA bacteremia cases were prevented during the seven years studied, or 34 cases annually. The cost of treating these anticipated 34 cases is estimated to have been $407,700 (the averted cost).

### The Overall Impact of the Intervention

Our analysis shows that the MRSA-directed intervention prevented 34 cases of bacteremia and 12 bacteremia-related deaths per year. When the annual cost of averted bacteremia ($407,700) and that of prevention ($208,100) are compared, the intervention resulted in an annual cost saving of $199,600, a saving to cost ratio of about two.

Sensitivity analysis: A sustained decrease in MRSA bacteremia cases of 54% or more is needed for the intervention to be cost saving, not taking into account lives saved.

If we consider a change of 15% in the percentage of time dedicated to the program by the ICP team, the total cost of prevention would increase from $202,300 to $214,000, a minimal amount.

## Discussion

Infections with multidrug-resistant bacteria are a public health concern internationally. These resistant bacteria increase the infection burden in hospitals, increase morbidity and mortality, and increase broad-spectrum antibiotic use. In several North European countries, nationwide interventions to prevent the spread of MRSA have been implemented successfully.[12] However, many other countries have not adopted such programs, mostly because of the increased workload and cost. Several cost-benefit analyses of MRSA prevention interventions have been published, the majority of which show that the benefits outweigh the costs. [13] Therefore, it is important to show similar effects of implementing these interventions within a variety of health care systems and cost considerations. The fact that our findings concur with those from different countries and different healthcare system adds to the generalizability of the results.

In most studies assessing MRSA cost, there is a clear distinction between patients who acquired MRSA in the hospital (nosocomial cases) and patients who were admitted with the infection; cost is calculated only for nosocomial cases because the hospital was “responsible” for those cases only. We argue that this approach to cost assessment is simplistic and wrong. Davis et al. assessed the rate of MRSA bacteremia in patients with nasal colonization. They found that 19% of patients admitted with MRSA colonization developed MRSA infection and 7.7% developed bacteremia during hospitalization, indicating that “nosocomial" MRSA infection is often a late manifestation of previous colonization.[14] Another study followed patients colonized with MRSA and found that bacteremia with the same strain appeared from one day to 14 months later, [15] demonstrating that colonization acquired in the current hospitalization can present as bacteremia on admission in a future hospitalization. Moreover, since many hospitals serve the surrounding community, patients tend to return to the same hospital. Thus, today's MRSA colonization will translate into future infection burden and costs to the same facility and vice versa, investment in prevention will reduce not only the costs of hospitalized patients, but will also decrease future admissions and costs. This was exemplified in our previous study in which we demonstrated that the MRSA prevention intervention resulted in a decrease over time in the percentage of MRSA carriers at admission.[10] Recently, an observational study from 112 Veterans Affairs medical centers demonstrated that after the implementation of a MRSA prevention bundle in 2007, the rate of hospital-acquired MRSA fell first, followed by a decrease in admission prevalence.[4] In this study, we included both the cost of patients who developed MRSA bacteremia during hospitalization and those admitted with MRSA bacteremia who were admitted to our hospital in the prior year. It is important to note that CO-HR cases were common and contributed significantly to the total cost of infections.

In most studies, including several mathematical models of costs and benefits of MRSA prevention, [16,17] data on the cost of prevention and the cost of MRSA infection were not collected from the same hospital.[18–20] This may have resulted in biased estimates, as health care facilities and local conditions may vary. In this study, the intervention cost, intervention effect, and infection cost were all calculated in the same hospital. This is both a strength as well as a limitation of our study. Although it is expected to have less bias and more precision, it may limit the generalizability of the findings.

The cost of MRSA bacteremia to patients admitted with the bacteremia was $14,300, comparable to the cost described in a study done in Spain of €11,044. [21] To estimate the cost of MRSA bacteremia, hospitalization costs were calculated to the individual laboratory test level. Interestingly, the greater part of the cost was attributable to fixed costs; the cost attributable to antibiotics was miniscule by comparison. Although MRSA BSI is an infectious disease and although some of the antibiotics used to treat these infections are considered expensive, the treatment of an MRSA patient extends far beyond antibiotics.

MRSA BSI not only prolonged hospitalization and increased expenses, but also led to increased mortality. The crude MRSA-attributable, in-hospital mortality in our patients was about 36%, both in patients hospitalized with the infection and in patients who acquired the infection during hospitalization (51% in cases and 16% mortality in controls). For hospital-acquired MRSA BSI, the average cost among those surviving at least 72 hours was more than twice the cost for survivors and non-survivors combined.

Our intervention reduced morbidity and mortality and was also cost saving. The saving-cost ratio was about two. A meta-analysis published in 2013 included 18 studies that calculated both costs and benefits. The mean save-cost ratio was 7.16 [13] and 15 of the 18 studies presented a ratio over one. Our ratio was lower than the mean, likely because we used a conservative estimate that included the prevention of bacteremia only and did not include the effect of the intervention on other MRSA infections, such as surgical site infections, pneumonia, or soft tissue. Since our intervention was aimed at preventing the spread of MRSA, it is very likely that the preventive effect extended to these infections as well.

This study had several limitations. One was the use of a single 70% MRSA BSI reduction value. This decrease was achieved at the end of a multifaceted intervention. As expected, some variability over time was evident. However, since 2007 the sustained decrease in bacteremia cases fluctuated from 60% to 76% (with 76% reduction in 2014). As shown in the sensitivity analysis, a sustained reduction over 54% was needed for the intervention to be cost saving, not taking into account lives saved. An additional limitation was that the prevention intervention included some different components over time. [10] Notably, the last period of the intervention was the most successful in reducing bacteremia cases (2007). Therefore, the cost of the elements included in the last period was used for the analysis. Moreover, this intervention has been used continuously in our hospital since. Another potential limitation is the possibility of missing confounders which would therefore be missed in the logistic regression.

Optimal long-term outcomes of prevention interventions can be seen in northern Europe [22], where interventions are country-wide. Our results show that MRSA-directed prevention can be highly effective and cost-saving in a high-incidence country, even when performed in a single institution. However, we hope our results will engender a country-wide intervention to limit the spread of MRSA and reduce its prevalence.
